# Reduced Mastication Is Associated With Dynapenia Markers in Patients With Cirrhosis: A Cross-Sectional Study

**DOI:** 10.1155/ijh/5842659

**Published:** 2025-05-05

**Authors:** Bruna Brunetto, Leonardo Saraiva, Sidia Maria Callegari-Jacques, Hérica Ferri, Helena Bernieri Lizott, Ricardo Valões, Sabrina Alves Fernandes, Lisia Hoppe, Fernando Fornari

**Affiliations:** ^1^Post Graduate Program in Dentistry, University of Passo Fundo, Passo Fundo, Rio Grande do Sul, Brazil; ^2^Department of Statistics, Federal University of Rio Grande do Sul, Porto Alegre, Rio Grande do Sul, Brazil; ^3^School of Medicine, University of Passo Fundo, Passo Fundo, Rio Grande do Sul, Brazil; ^4^Hepatology Department, São Vicente de Paulo Hospital, Passo Fundo, Rio Grande do Sul, Brazil; ^5^Post Graduate Program in Hepatology, Federal University of Health Sciences of Porto Alegre, Porto Alegre, Rio Grande do Sul, Brazil

**Keywords:** cirrhosis, dynapenia, mastication, muscle, sarcopenia

## Abstract

**Background and Aim:** Dental diseases are common in patients with cirrhosis. In these patients, reduced mastication might interfere with protein intake and contribute to malnutrition. We addressed the relationship between reduced mastication and dynapenia in patients with cirrhosis.

**Methods:** This cross-sectional study involved patients with cirrhosis treated in a Brazilian center. Trained dentists performed oral examinations and tested the patients for nutritional parameters such as handgrip strength (HGS) and gait speed test (GST). Reduced mastication was presumed when a patient had molar edentulism (≥ 3 teeth), bad dental occlusion, or ill-fitting denture. Associations between mastication status and malnutrition were evaluated using multivariate linear regression analysis for continuous measures and adjusted prevalence ratio (PR (95% confidence interval)) for binary measures.

**Results:** We included 149 patients with cirrhosis (60 ± 13 years old, 76% men, 64% Child A, 60% due to alcoholism only). Reduced mastication affected 107 patients (72%), low muscle strength (decreased HGS) occurred in 45 (30%), and decreased GST was observed in 58 (41%, among 143 patients able to walk). Thirty-one out of 143 (22%) presented decreased HGS and GST, characterizing dynapenia. Reduced mastication was associated either with decreased HGS [PR = 2.28 (1.08–4.81), *p* = 0.030; reduced mastication decreases the HGS mean by 12.5 kg for men (*p* < 0.001) and 8.1 kg for women (*p* = 0.065)] or with decreased GST [PR 1.97 (1.09–3.55), *p* = 0.024; reduced mastication increased the time of GST by 1.1 s on average (*p* = 0.005)], adjusting for age, alcoholic etiology, and Child–Pugh classification.

**Conclusions:** Reduced mastication is associated with dynapenia markers in patients with cirrhosis. Further studies are needed to assess whether oral rehabilitation can change the curse of malnutrition in this population.

## 1. Introduction

Liver cirrhosis is the outcome of a long period of inflammation, resulting in fibrosis, vascular rearrangement, and liver dysfunction, which can lead to transplantation or death in a few years [1, 2]. These hepatic changes usually evolve with clinical complications including ascites, encephalopathy, and variceal bleeding, related to triggering factors such as sarcopenia and infections [2, 3]. In 2019, cirrhosis caused 2%–4% of global deaths [4]. Nowadays, its main etiologies are alcohol use disorder, Hepatitis C, Hepatitis B, and nonalcoholic fatty liver disease [1].

Patients with cirrhosis often present oral conditions, particularly periodontitis [5]. Combined with poor hygiene, caries, and xerostomia, they can lead to edentulism [6]. Substantial dental injuries, especially in patients with alcoholic cirrhosis [7], might compromise the masticatory function and contribute to malnutrition.

Mastication patterns can be diagnosed with a detailed dental examination. Recent studies suggest that reduced mastication due to edentulism, particularly the loss of molar teeth responsible for food trituration, is associated with upper digestive conditions, such as dysphagia, gastroesophageal reflux disease, and dyspepsia [8–10]. Bad dental occlusion and ill-fitting dentures can also be objective criteria to predict masticatory dysfunction [9, 10].

Liver cirrhosis can be complicated by the clinical condition of sarcopenia, known as progressive loss of volume and functionality of skeletal muscle mass [11], and by dynapenia, which is related to decreased muscle strength, compromising physical performance [12]. These two clinical conditions configure and enhance malnutrition, which directly impacts the prognosis of cirrhosis. Sarcopenia affects between 40% and 70% of patients with cirrhosis [13–15] and is related to increased pre- and postliver transplant morbidities [16, 17]. The loss of muscle mass becomes more pronounced with the worsening of liver reserve and is an independent risk factor for cirrhosis-related death [17, 18]. Although an association between poor oral health and malnutrition was recently suggested [19], there is no attention to this association in patients with cirrhosis.

We hypothesized that reduced mastication is prevalent in patients with liver cirrhosis, and this condition may be associated with dynapenia or sarcopenia due to compromised food intake and, consequently, the emergence of malnutrition. We, therefore, addressed the relationship between reduced mastication and dynapenia in patients with cirrhosis.

## 2. Materials and Methods

STROBE statement recommendations were followed in the design and reporting of this study.

### 2.1. Design, Setting, and Participants

A cross-sectional study occurred in Passo Fundo (Brazil) between September 2022 and January 2024. We recruited patients treated in a reference center for liver diseases, either from the outpatient unit or hospital setting (Hospital São Vicente de Paulo, in Passo Fundo, Brazil). Participants were adult patients with cirrhosis diagnosed by the research team hepatologists (RV and LH). Exclusion criteria were the inability to collaborate due to encephalopathy and hepatic transplantation. All participants signed an informed consent before entering the study, which followed the rules of the Helsinki Declaration, with approval by the local Research Ethics Committee (number: 5.619.521).

### 2.2. Clinical Examination

Trained dentists (BB, LS, and HF) interviewed the patients and performed clinical examinations. Demographic and medical data included age, sex, and cirrhosis details, such as disease confirmation, etiology, Child–Pugh classification, ascites, encephalopathy, and hepatocellular carcinoma. We assessed dynapenia and sarcopenia using handgrip strength (HGS), gait speed test (GST), and calf circumference (CC). Oral examination addresses the number and condition of teeth, dental prosthesis, facial pattern, and dental occlusion [10]. Patients replied to a questionnaire for assessment of xerostomia, as described elsewhere [20].

### 2.3. Assessment of Malnutrition

**Figure 1:**
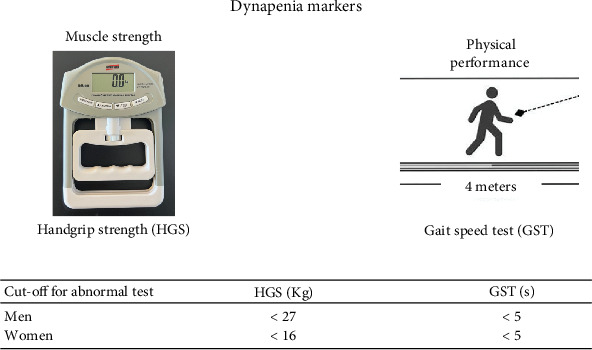
Markers of dynapenia.

We assessed malnutrition using conventional criteria (Figure 1) [21]: HGS as a marker of muscle strength, GST for physical performance, and CC representing muscle mass. HGS was measured using a commercially available digital dynamometer (calibrated for 90 kg, Instrutherm, São Paulo, Brazil). We repeated the test twice using the dominant arm, considering the best rate. The cut-off for identification of decreased HGS was < 27 kg for men and < 16 kg for women [22, 23]. GST consisted of a timed 4-m usual walking speed. A single cut-off gait speed of ≤ 0.8 m/s (or 5 s) was considered abnormal [24, 25]. We used a standard measuring tape to measure the CC in the middle of the leg. Low muscle quantity was diagnosed with a CC lower than 35 cm in men and lower than 34 cm in women 30 [26].

### 2.4. Mastication

**Figure 2:**
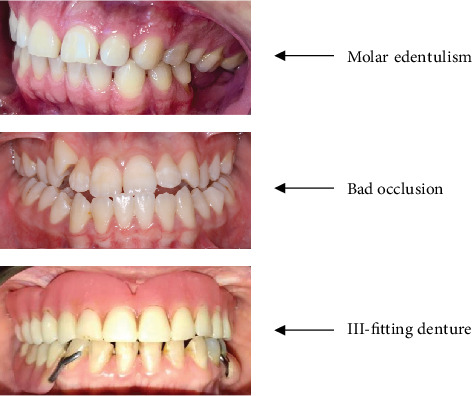
Criteria used for characterization of reduced mastication.

We categorized mastication as reduced vs. regular. These categories were determined by trained dentists (BB, LS, HF) after oral examination, using three objective criteria (Figure 2): (i) dental occlusion, classified as ideal, either with natural teeth or adequate prosthetic rehabilitation, mildly compromised by a slight change in occlusion balance, and severely compromised occlusion by unfavorable facial pattern, ill-fitting dentures, and tooth loss without rehabilitation, (ii) dental prostheses classified as perfect and ill-fitting, and (iii) tooth loss, including number and dental type. We asked for a self-report of reduced mastication for any reason but did not consider the answer as a criterion due to potential influences of hepatic encephalopathy. The participants had reduced mastication in the presence of at least one of the following criteria: severely compromised occlusion, ill-fitting dentures, and loss of at least three molar teeth without rehabilitation.

### 2.5. Variables

Reduced mastication (qualitative, yes/no) was the independent variable, whereas dynapenia/sarcopenia was the outcome. Dynapenia markers were HGS (quantitative and qualitative (decreased/normal)) and GST (quantitative and qualitative (decreased/normal)). Sarcopenia included HGS, GST, and CC (quantitative and qualitative (decreased/normal)). Potential confounders for the association between mastication and malnutrition were age, sex, Child–Pugh classification (A, B, or C), etiology of cirrhosis (alcohol/other causes), xerostomia (qualitative, yes/no), oral hygiene (qualitative, good/poor), and periodontal disease (yes/no).

### 2.6. Sample Size and Statistical Analysis

We estimated the sample size based on HGS measures. A hundred patients (75 individuals with reduced mastication and 25 with regular mastication) were adequate to identify a minimum difference of 8 kg in HGS between the two groups, with 0.9 power and 0.05 as the threshold for statistical significance. In this calculation, we considered standard deviation (SD) = 10 kg (based on Wang et al. [27]) and used the software PSS-Health (<https://hcpa-unidade-bioestatistica.shinyapps.io/PSS_Health/>).

We described continuous data using mean ± SD and categorical variables with absolute frequencies and percentages. Student's *t*- or Mann–Whitney *U* tests compared groups of continuous variables according to their distribution, and Pearson's exact chi-square test analyzed categorical variables.

To analyze the association between reduced mastication and quantitative measures of malnutrition, we used generalized linear models (GZLMs) with model-based estimators for the covariance matrix, gamma distribution, log link for HGS and GST, and normal distribution with identity link for CC. When adjusting for confounding, we evaluated potential covariables based on theoretical considerations, as well as on the observed statistical association of each covariable with both reduced mastication and the outcomes, and the collinearity among covariables. The resulting confounders were age dichotomized into < 65/65 or more years due to the lack of linearity-of-effect assumption, etiology of cirrhosis (alcohol vs. others), and Child–Pugh classification (A vs. B and C). Males and females were analyzed separately when evaluating the HGS scores.

To estimate the association between reduced mastication and the three binary definitions of malnutrition (yes/no: decreased HGS, CC, and GST), we used Poisson's regression models with robust estimators of covariance to obtain crude and adjusted prevalence ratios (PRs) and respective 95% confidence intervals. In these analyses, age (< 65/65 or more), alcoholic etiology, and Child–Pugh classification (A vs. B and C) were used as covariables. We used SPSS v.18 for the analyses and considered 0.05 as the limit for statistical significance in all tests.

## 3. Results

### 3.1. Participants

**Figure 3:**
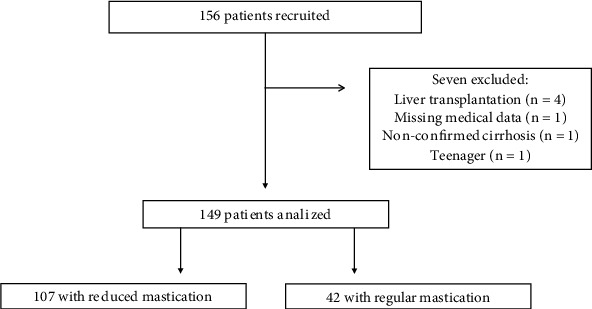
Participant flow chart.

A total of 156 patients agreed to participate (Figure 3). We excluded seven participants due to liver transplantation (*n* = 4), missing medical data (*n* = 1), nonconfirmed cirrhosis (*n* = 1), and a 13-year-old teenager (*n* = 1). Among 149 analyzed patients (60 ± 13 years old, 76% men, 64% Child–Pugh A, 60% due to alcoholism only), 107 had reduced mastication (72%), and 42 (28%) had regular mastication. We examined 124 (83%) on an outpatient basis and 25 patients (17%) during hospitalization.

Patients in the reduced mastication group were approximately 10 years older than patients with regular chewing (Table 1), and men corresponded to three-quarters in both groups. Patients with reduced mastication had a higher proportion of cirrhosis exclusively due to alcohol abuse than patients with regular chewing. The rates of Child–Pugh A (vs. B and C), ascites, hepatic encephalopathy, and hepatocellular carcinoma did not differ according to mastication status.

### 3.2. Oral Conditions and Mastication Status

Xerostomia occurred in a third of patients with reduced mastication and a fourth of patients with regular chewing, with no statistical difference between groups (Table 2). In both groups, periodontal disease was equally present, affecting a fourth of the participants. Bad oral hygiene was significantly more prevalent in patients with reduced than in those with regular chewing.

Among the 107 participants with reduced mastication, the numbers observed for the criteria for its characterization were (i) bad dental occlusion (*n* = 81 patients), (ii) loss of molar teeth without rehabilitation (*n* = 41), and (iii) molar edentulism with the use of ill-fitting dentures (*n* = 66).

### 3.3. Dynapenia Markers

Probable dynapenia indicated by low muscle strength (decreased HGS) affected 45 out of 149 patients (30%). Decreased HGS was more frequent in patients with reduced mastication than those with regular chewing (36% vs. 14%, *p* = 0.009, Table 3). As HGS scores differed significantly between men and women, we performed separate analyses by sex. Lower HGS values were associated with reduced mastication in both sexes. The HGS mean for males with reduced mastication was 29.4 kg compared to 42.6 kg for males with regular mastication (difference 13.2 kg, *p* = 0.001). In females, the means were 19.7 and 32.0 kg, respectively (difference 12.3 kg, *p* = 0.009). There was no difference between sex for CC and GST scores.

The parameter for muscle mass (CC) did not differ between patients with reduced mastication and those with regular mastication, either for the categorical classification of decreased CC (*p* = 0.490) or using the quantitative measure of CC (*p* = 0.451, Table 3). For GST evaluation, we excluded six patients who did not walk (all with reduced mastication). GST lasted longer in patients with reduced mastication (mean difference 1.2 s, *p* = 0.005), and more of these patients showed decreased GST than patients with regular mastication (*p* = 0.003). Confirmed dynapenia (decreased HGS and decreased GST) occurred in 31 out of 143 patients (22%). We observed a higher prevalence of confirmed dynapenia in patients with reduced mastication than in those with regular chewing (26% vs. 12%).

### 3.4. Crude and Adjusted Association Between Reduced Mastication and Dynapenia Markers

We analyzed the association between reduced mastication and dynapenia measured by HGS and GST (as qualitative and quantitative parameters), using regression models to adjust for potential confounder effects of age, alcohol etiology of cirrhosis, and Child–Pugh classification. In the univariate analyses of the qualitative parameters (Table 4), reduced mastication was associated either with decreased HGS [PR 2.55 (95% CI 1.17–5.58), *p* = 0.019] or decreased GST [PR 2.26 (1.23–4.18), *p* = 0.009]. These associations did not change when adjusting for age, alcoholic etiology, and Child–Pugh classification both for decreased HGS [PR 2.28 (1.08–4.81), *p* = 0.030] and decreased GST [PR 1.97 (1.09–3.55), *p* = 0.024]. For both parameters, the adjusted prevalence of dynapenia doubles if the patient with cirrhosis has reduced mastication.

For quantitative dynapenia measures, the HGS means adjusted for age, alcoholic etiology, and Child–Pugh classification were 26.6 kg for men with reduced mastication and 39.1 kg for men without this characteristic (mean difference = 12.5 kg, *p* < 0.001). The adjusted means for women were 19.7 kg and 27.8 kg, respectively (difference = 8.1 kg, *p* = 0.065). Reduced mastication increased the time of GST by 1.2 s on average (*p* = 0.001). The adjustment for age, alcoholic etiology, and Child–Pugh classification did not change this result (average difference 1.1 s, *p* = 0.005).

## 4. Discussion

Cirrhosis is a relevant, costly, multisystem disease in which the damaged liver interacts with the entire body, resulting sooner or later in complications and death [2, 3]. The present study addressed a new “liver–body axis” focused on the relationship between chewing and nutrition in patients with cirrhosis. Reduced chewing was recently recognized as a risk factor for upper gastrointestinal conditions [8–10] and is an established obstacle to nutrient digestion and absorption [28]. Given the high risk for dental injuries in patients with chronic liver disease, particularly in alcohol abusers [7], we hypothesized that patients with cirrhosis are of particular concern for mastication dysfunction and malnutrition.

In this cross-sectional study involving 149 patients with cirrhosis, we found that reduced chewing was associated with markers of dynapenia, specifically HGS and GST. HGS measured with a hand dynamometer is the gold standard for diagnosing low muscle strength [21, 22], which characterizes probable dynapenia. In the present study, when adjusting for age, alcoholic etiology for the cirrhosis, and Child–Pugh classification, reduced mastication decreased the HGS mean by 12.5 kg in men. A reduction of 8.1 kg was observed in women, with borderline *p* value, probably due to a small number of females in the study (*n* = 36). GST was the second dynapenia marker associated with reduced mastication. Chewing dysfunction increased the time of GST by 1.1 s on average. When abnormal, this marker of physical performance is negatively associated with survival in aging [25]. From a different perspective, we estimated that the prevalence of reduced HGS and reduced GST doubles for patients with mastication problems (PR 2.28 and 1.97, respectively).

Although not studied, the likely mechanism for this association is nutritional impairment secondary to masticatory dysfunction, potentially compromising food intake in quantity and quality. A link between tooth loss and malnutrition occurs in adults over 50 [29]. A recent study showed that dysphagia, which can be related to chewing problems [9], was prevalent in patients with chronic liver disease and was associated with sarcopenia, malnutrition, and decreased health-related quality of life [30]. Since protein intake depends on consuming solid foods such as meat, an adequate masticatory function is necessary for diets containing such consistencies [28, 31]. Whether masticatory dysfunction provokes different effects between protein malnutrition and calorie intake deserves additional studies. On the other hand, the reverse causality can play a role. Malnutrition might cause masticatory dysfunction due to oral sarcopenia, as already demonstrated in aging [32]. Studies are needed to evaluate oral sarcopenia in patients with cirrhosis.

Expert opinion advocates that the assessment of muscle strength and physical performance can help the identification of malnutrition in clinical practice [33]. However, muscle mass assessed with CC was not associated with reduced mastication. Controversies exist about the accuracy of CC as a muscle mass marker [34, 35]. Fluid retention in the legs of patients with cirrhosis presenting ascites might bias the CC measurement. Further studies can address the relationship between reduced mastication and sarcopenia with tomographic muscle quantification.

Our study showed that confirmed dynapenia affected 23% of participants, most of them with compensated cirrhosis. Evidence supports a connection between dynapenia and fatty liver disease, in which muscle quality relates to *β*-cell function [36, 37]. A meta-analysis described that the overall prevalence of sarcopenia in patients with cirrhosis is 37.5%, particularly in males, alcohol-related liver disease, and decompensated cirrhosis [38]. In adults with sarcopenia, sarcopenic obesity, or decompensated cirrhosis, a recent guideline recommended a high-protein diet [39], for which is expected a preserved masticatory function.

We studied patients with cirrhosis, mainly men in their six decades, with alcoholism as the principal etiology [40]. Two-thirds had compensated cirrhosis (Child–Pugh Class A), and the majority (83%) were evaluated in an ambulatory setting, while less than 10% presented cirrhosis in a terminal stage (Child–Pugh C). Reduced mastication occurred in 72% of these patients and was related to older age (~10 years) and a higher prevalence of alcoholism. Yet periodontal disease affected only a quarter of participants, likely due to the high prevalence of edentulism in this population. The distribution of Child–Pugh classification, ascites, hepatic encephalopathy, and hepatocellular carcinoma did not differ regardless of the mastication status despite moderate to massive ascites being numerically higher in patients with reduced mastication. These characteristics agree with epidemiological studies involving patients with chronic liver disease [4].

As far as we know, this is the first study addressing the relationship between chewing and malnutrition in patients with cirrhosis, involving a research team with hepatologists and dentists trained to diagnose chewing patterns and malnutrition markers. Reduced chewing was estimated using criteria developed and published elsewhere [8–10], adapted to patients with cirrhosis. We excluded the self-report of reduced mastication to avoid the potential influence of hepatic encephalopathy, found in nearly 20% of patients. Hence, we used only objective data to classify mastication. The criterion of molar edentulism was set in three or more teeth losses, irrespective of molar location, to facilitate the diagnosis in medical practice. We believe the findings of this study are generalizable to liver cirrhosis patients worldwide.

This study has limitations and strengths. Although the sample estimation indicated a lower number of patients (100 patients) than what was effectively studied, our sample of 149 patients generated some associations with borderline significance that could demand more patients. We did not evaluate hypogeusia, potentially involved in reduced food intake in patients with cirrhosis [41]. The tools for malnutrition assessment did not include muscle quantification with computed tomography, considered the gold standard for muscle mass evaluation [11]. Instead, we included accessible, low-cost, scientifically approved markers such as HGS and GST [21]. Due to multicollinearity, the estimates were adjusted for age, alcoholic etiology for cirrhosis, and Child–Pugh classification. We had the caution to address other potential confounders of oral conditions, such as xerostomia and periodontal disease, as well as critical medical information before the decision concerning adjustment.

In conclusion, we performed a cross-sectional study in patients with cirrhosis to address the relationship between chewing and malnutrition. In this study group, reduced mastication was highly prevalent and was associated with dynapenia markers, either HGS or GST. Whether oral rehabilitation can change the curse of dynapenia in patients with cirrhosis needs further elucidation in well-conducted interventional studies.

## Figures and Tables

**Table 1:** Demography and medical conditions (*n* = 149) according to mastication status.

	**Reduced mastication (** **n** = 107**)**	**Regular mastication (** **n** = 42**)**	**p** ** value**
Age in years, mean ± SD	62.3 ± 9.7	52.5 ± 16.9	0.001^b^
Men, *n* (%)	81 (76%)	32 (76%)	0.999^c^
Cirrhosis etiology, *n* (%)			
Alcohol^a^	80 (75%)	26 (62%)	0.089^c^
Causes other than alcohol	27 (25%)	16 (38%)	
Child–Pugh class, *n* (%)			0.342^c^
A	66 (62%)	30 (71%)	
B and C	41 (38%)	12 (29%)	
Ascites, *n* (%)⁣^∗^	53 (50%)	23 (55%)	0.590^c^
Encephalopathy, *n* (%)			0.150^c^
Covert	85 (79%)	38 (90%)	
Overt	22 (21%)	4 (10%)	
HCC^d^, n (%)	16 (15%)	3 (7%)	0.278^c^

⁣^∗^15% of patients with reduced mastication had moderate to massive ascites vs. 9% of patients with regular mastication (*p* = 0.438).

^a^Uniquely and combined with other causes.

^b^Student's *t*-test.

^c^Exact chi-square.

^d^Hepatocellular carcinoma.

**Table 2:** Oral conditions (*n* = 149) according to mastication status.

**N** ** (%)**	**Reduced mastication (** **n** = 107**)**	**Regular mastication (** **n** = 42**)**	**p** ** value**^**d**^
Xerostomia	37 (35%)	11 (26%)	0.340
Periodontal disease	28 (26%)	10 (24%)	0.837
Poor oral hygiene^a^	59 (55%)	3 (7%)	< 0.001
Dental occlusion			^e^
Optimal	4 (4%)	18 (43%)	
Acceptable	22 (21%)	24 (57%)	
Bad	81 (75%)	0 (0%)	
Edentulism ≥ 3 molars	106 (99%)	9 (21%)^b^	^e^
Dentures^c^			^e^
Not rehabilitated	41 (38%)	—	
Anterior perfect	0 (0%)	9 (50%)	
Posterior perfect	0 (0%)	9 (50%)	
Ill fitting	66 (62%)	0 (0%)	

^a^Vs. normal/regular hygiene.

^b^Nine patients with dental rehabilitation after adequate prosthetic treatment.

^c^Anterior means teeth other than the molars, posterior means molar teeth, and ill-fitting denture means bad occlusion and or poorly adapted to the supporting tissue.

^d^Exact chi-square.

^e^Non comparable variables due to criteria entry.

**Table 3:** Malnutrition parameters in 149 participants according to mastication status.

**N** ** (%)**	**Reduced mastication (** **n** = 107**)**	**Normal mastication (** **n** = 42**)**	**p** ** value**
Handgrip strength (kg)			
Males: quantitative, mean ± SD (*n*)	29.4 ± 12.2 (81)	42.6 ± 18.4 (32)	0.001^b^
Females: quantitative, mean ± SD (*n*)	19.7 ± 8.3 (26)	32.0 ± 18.4 (10)	0.009^b^
Decreased, *n* (%)	39 (36%)	6 (14%)	0.009^c^
Calf circumference (cm)			
Quantitative, mean ± SD	38.1 ± 4.8	37.5 ± 4.8	0.451^b^
Decreased, *n* (%)	19 (18%)	10 (24%)	0.490^c^
Gait speed test (s)^a^			
Quantitative, mean ± SD	5.8 ± 2.5	4.6 ± 2.1	0.005^b^
Decreased, *n* (%)	49 (49%)	9 (21%)	0.003^c^

^a^ *N* = 143 (six patients with reduced mastication could not walk).

^b^Student's *t*-test.

^c^Exact chi-square.

**Table 4:** Crude and adjusted measures of association between reduced mastication and dynapenia defined as decreased HGS (handgrip strength) and decreased GST (gait speed test).

**Outcome**	**Univariate analysis**		**Multivariate analysis**	
	**p**	**Crude PR** ** (95% CI)**	**p**	**Adjusted PR** ^**a**^ ** (95% CI)**
Reduced HGS	0.019	2.55 (1.17–5.58)	0.030	2.28 (1.08–4.81)
Decreased GST	0.009	2.26 (1.23–4.18)	0.024	1.97 (1.09–3.55)

^a^Prevalence ratio adjusted by age (< 65/65 or more years), alcoholic etiology for cirrhosis (yes/no), and the Child–Pugh classification (A vs. B and C).

## Data Availability

The data that support the findings of this study are available from the corresponding author upon reasonable request.
